# Transcatheter Aortic Valve Replacement Restoring Candidacy for Liver Transplant in Patients With Cirrhosis

**DOI:** 10.14309/crj.0000000000001102

**Published:** 2023-08-17

**Authors:** Mirna Kaafarani, Omar Shamma, Syed-Mohammed Jafri

**Affiliations:** 1 Wayne State University School of Medicine, Detroit, MI; 2 Department of Gastroenterology and Hepatology, Henry Ford Health System, Detroit, MI

## Abstract

Guidelines for preoperative workup for an orthotopic liver transplant often rule out patients with severe aortic stenosis as transplant candidates. This case illustrates the potential of transcatheter aortic valve replacement (TAVR) as a bridge for liver transplants in cirrhotic patients with severe aortic stenosis. The 1-year and 2-year post-liver transplant follow-ups showed no complications in the patient's prosthetic aortic valves, and graft survival was 100% with no evidence of rejection. Notable post-transplant recovery involved medical complications that were not related to the liver function or surgical procedure.

## INTRODUCTION

Severe aortic stenosis (AS) has been suggested to be a strong predictor for perioperative mortality in noncardiac surgeries with an increased risk of cardiovascular complications.^1–3^ AS is classified as severe when the transaortic velocity is ≥4.0 m/s or the aortic valve area is <1.0 cm^2^ or a mean gradient of ≥40 mm Hg.^4–6^ When considering orthotopic liver transplant (OLT) in cirrhotic patients with concomitant severe AS, the current guidelines for preoperative workup recommend cardiac testing that classically rule out these patients.^7^ Similarly, surgical aortic valve replacement in the setting of the hemodynamic changes associated with liver cirrhosis has been shown to increase mortality, complications, cost, and length of hospital stay.^8^ The relatively novel transcatheter aortic valve replacement (TAVR) has been approved for both high and low-risk patients with severe AS and is growing as the treatment of choice in those populations.^9,10^

## CASE REPORT

In 2018, a 63-year-old man with a history of alcoholic cirrhosis with hypertension and esophageal varices was referred to our unit for evaluation of the potential role of liver transplant. He was diagnosed 6 years earlier when he was living out of state with cirrhosis. He never followed up for proper hepatic care and disclosed that he continued to drink heavily. His condition progressed without any notable symptoms until he first presented with ascites that required 2 large volume paracenteses between 2016 and 2017. In addition to his liver disease, the patient's medical history also included a semiemergent open-heart replacement of the aortic valve, in 2012, after an echocardiogram incidentally demonstrated a bicuspid aortic valve with severe stenosis. His prosthetic mechanical aortic valve had degenerated leading to the recurrence of AS over the years. This recurrence of AS disqualified the patient from being considered for an OLT, and his progression of cirrhosis rendered a second surgical aortic valve replacement too high of a risk hemodynamically.

In 2017, the hospital performed an echocardiogram to ascertain how far the AS had progressed, and it showed a peak and median aortic valve gradients of 67.7 mm Hg and 38.3 mm Hg, respectively. They decided to treat it with a transfemoral mechanical valve-in-valve TAVR. No complications were reported. After this procedure, the patient transferred his care to our clinic. We assessed his aortic gradients with a post-TAVR echocardiogram with the hope of restored candidacy for an OLT. The post-TAVR echocardiogram showed decreased peak and median aortic gradients of 51.4 and 24.5 mmhg, respectively, with a continued preserved ejection fraction. Our multidisciplinary team deemed that the patient had an acceptable cardiovascular risk to proceed and underwent a standard OLT from a deceased donor with a duct vasovasostomy (Table 1).

**Table 1.** A comparison of echocardiogram parameters of the aortic valve before and after a TAVR procedure and after OLT

	Before TAVR	After TAVR	After OLT
Aortic valve peak gradient (mm Hg)	16.9	12.2	16.6
Aortic valves mean gradient (mm Hg)	8.4	5.8	9.8
Left ventricular ejection fraction (%)	61	46	55
Aortic valve area, Vmax (cmÂ^2^)	2.22	1.53	3.12

The post-TAVR parameters were measured 2 days after the procedure. The post-OLT parameters were measured 2 months after the transplant.

OLT, orthotopic liver transplant.

The postoperative course was significant for the development of stroke-like symptoms with right facial droop and pronator drift. Brain magnetic resonance imaging showed chronic ischemic changes, and abdominal computed tomography revealed 74% stenosis of right internal carotid artery. Antiplatelet agent and statin therapies were begun, and symptoms resolved within 24 hours. In addition, a duplex of neck vessels demonstrated right internal jugular and subclavian thrombus, and the patient was started on a heparin drip as a bridge to coumadin. He then developed an intraperitoneal hematoma with a drop in hemoglobin requiring 2 units of packed red blood cells. An episode of hematoma below the allograft and right kidney, likely secondary to anticoagulation during transient ischemic attack in the setting of the 74% stenosed right internal carotid artery, was successfully drained percutaneously. Finally, the patient also developed rapid atrial fibrillation/flutter after the operation on day 1. The patient was treated with beta blockers and symptoms improved.

There was patient and graft survival at 1 year and 2 years after transplant with no evidence of rejection. Cardiac evaluation of 1-month, 3-month, and 1-year post-liver transplant showed no complications in the function of or related to the prosthetic aortic valve in valve. His liver function laboratory test results normalized a month out of his OLT and have stayed within a healthy range since with the most recent alanine aminotransferase, aspartate aminotransferase, and albumin of 13 IU/L, 14 IU/L, and 3.9 g/dL, respectively.

## DISCUSSION

AS is one of the most common valve pathologies of the heart in developing countries with a prevalence of 3.4% in patients older than 75 years.^11^ The expansion of TAVR indications to both high and low-risk candidates is revolutionizing the care of these patients with an approximated additional 270,000 TAVR candidates in the European Union and in Northern America annually.^12^ Likewise, the prevalence of alcoholic fatty liver disease and advanced fibrosis is also on the rise in the United States.^13^ In this setting, we wanted to contribute to the currently scarce compilation of case reports that illustrates the effectiveness that the TAVR procedure has on relieving the pretransplantation risks of AS in high-risk surgical aortic valve replacement patients.

Current literature briefly introduces the idea of TAVR successfully restoring candidacy for OLT. In 2019, Levy et al described 3 cases of patients in their sixth and seventh decades with hepatocellular carcinoma and severe AS that were all initially ineligible and then went through successful liver transplants after TAVR procedures. The cases were reported to have good liver function and no heart-related major adverse events, but their follow-ups were relatively shorter ranging from 6 to 10 months compared with our 24-month follow-up.^14^ In 2019, Kaliamoorthy et al described 2 cases of patients with infective endocarditis-induced acute aortic regurgitation similar to our case who underwent TAVR and then a subsequent living donor liver transplant successfully.^15^

When discussing the potential for TAVR, the concern of the safety of this cardiac procedure in a hemodynamically vulnerable cirrhotic patient population must also be brought to light. A recent 2021 investigation studied the 30-day and 1-year post-TAVR recovery course of over 300 patients with end-stage liver and/or kidney disease in comparison with a population without those comorbidities. Caughron et al concluded that the mortality at discharge and at 30 days was similar in both groups, suggesting no increased risk initially but with an upward trend of mortality at 1 year after TAVR.^16^ Ultimately, this case suggests the potential TAVR has at overcoming the cardiac barriers that are imposed on a successful OLT.

## DISCLOSURES

Author contributions: M. Kaafarani wrote the manuscript and is the author guarantor. O. Shamma edited the manuscript. SM Jafri edited the manuscript and revised the manuscript for intellectual content. SM Jafri is the article guarantor.

Financial disclosure: None to report.

Previous presentation: This case report was presented at the ACG Annual Scientific Meeting; October 25, 2022; Charlotte, North Carolina, and was awarded a Presidential Award.

Informed consent could not be obtained for this case report. All identifying information has been removed.
